# Prenatal diagnosis and molecular cytogenetic identification of small supernumerary marker chromosomes: analysis of three prenatal cases using chromosome microarray analysis

**DOI:** 10.18632/aging.202220

**Published:** 2020-12-09

**Authors:** Huili Xue, Xuemei Chen, Min Lin, Na Lin, Hailong Huang, Aili Yu, Liangpu Xu

**Affiliations:** 1 Department of Fujian Provincial Key Laboratory for Prenatal Diagnosis and Birth Defect, Fujian Maternity and Child Health Hospital, Affiliated Hospital of Fujian Medical University, Fuzhou 350001, Fujian, P.R. China; 2 Reproductive Medicine Center, Fujian Maternity and Child Health Hospital, Affiliated Hospital of Fujian Medical University, Fuzhou 350001, Fujian, P.R. China

**Keywords:** small supernumerary marker chromosome, single nucleotide polymorphism array, fluorescence in situ hybridization, isodicentric Y chromosome, ring X chromosome

## Abstract

Small supernumerary marker chromosomes cannot be accurately identified by G-banding, and the related phenotypes vary greatly. It is essential to specify the origin, size, and gene content of marker chromosomes using molecular cytogenetic techniques. Herein, three fetuses with *de novo* marker chromosomes were initially identified by G-banding. Single nucleotide polymorphism array and fluorescence *in situ* hybridization were performed to characterize the origins of the marker chromosomes. The karyotypes of the three fetuses were 47,XY,+mar, 46,X,+mar[32]/45,X[68], and 45,X[62]/46,X,+mar[9]. In case 1, the karyotype was confirmed as 47,XY,+ idic(22)(q11.2). Therefore, the sSMC originated from chromosome 22 and was associated with cat eye syndrome. In case 2, the marker chromosome derived from ring chromosome X, and the karyotype was interpreted as 45,X[68]/46,X,+r(X)(p11.1q21.31)[32]. Meanwhile, the karyotype of case 3 was defined as 45,X[62]/46,X,idic(Y)(q11.2) and the marker chromosome originated from chromosome Y. Case 1 continued the pregnancy, whereas the other two pregnancies underwent elective termination. The detailed characterization of marker chromosomes can facilitate informed decision making, prevent uncertainty, and provide proper prognostic assessments. Our findings emphasize the importance for combining cytogenetic and molecular genetic techniques in marker chromosome characterization.

## INTRODUCTION

Small supernumerary marker chromosomes (sSMCs) are extra chromosomes with structural abnormalities that are morphologically identifiable, and are generally equal to or smaller in size than chromosome 20 in the same metaphase karyotype, however, the origin and characteristics of sSMCs are not recognized by traditional chromosome banding techniques [1]. The detection rate of sSMCs in fetuses was reported as 0.08% via invasive prenatal diagnosis and 0.20% via anomaly ultrasound [2]. Fluorescence *in situ* hybridization (FISH) is an effective method for identifying sSMCs, particularly in mosaic chromosome abnormalities, however, selecting a suitable probe can prove challenging when the source of the fragment is unknown [3]. Hence, prenatal detection of sSMC currently poses significant challenges for obstetricians.

Once an sSMC is identified in a fetus, further molecular cytogenetic testing is required and particular attention must be paid to ultrasonography findings. Chromosome microarray analysis (CMA) is routinely employed prenatally due to their ability to detect copy number variations (CNVs) and uniparental disomy (UPD), particularly in defining sSMCs [4]. In the present study, Single nucleotide polymorphism (SNP) array and FISH were used to successfully characterize the chromosomal origin of sSMCs in three prenatal cases. Herein, we investigated the prenatal molecular cytogenetic diagnosis of three fetuses carrying sSMCs derived from chromosomes 22, X, and Y. FISH and CMA techniques were combined to understand the relationship between each sSMC and the resulting phenotype, and to accurately evaluate the prognosis of the three fetuses, which chromosome karyotype analysis of prenatal amniotic fluid is incapable of achieving.

## RESULTS

### Karyotype, CMA, and FISH results

**Figure 1:**
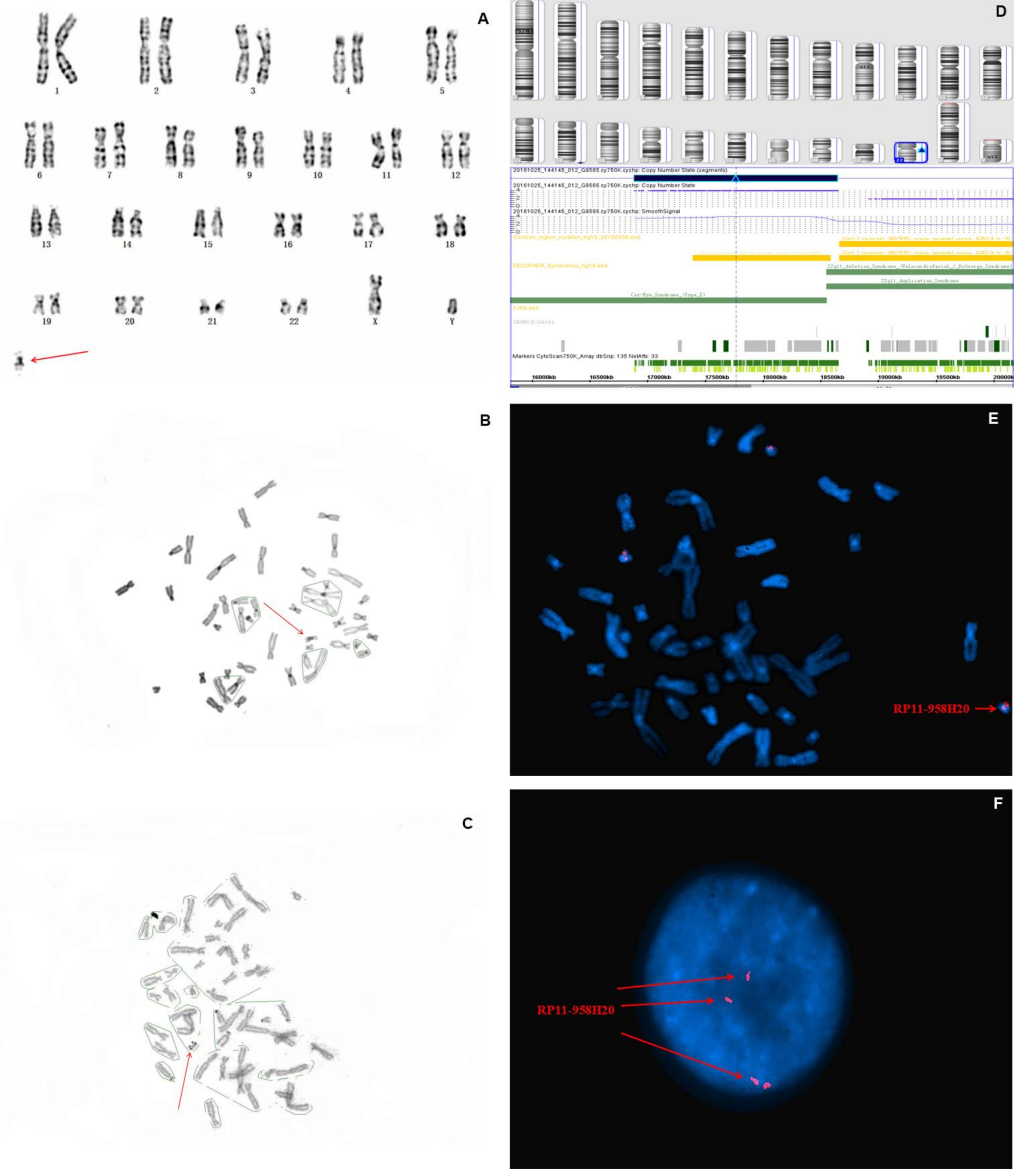
**Karyotype, CMA, and FISH analyses results for fetus 1.** The red arrow identifies sSMC. (**A**) G-banding, and (**B**) C-banding identified the marker as a pseudoisodicentric chromosome. (**C**) N-banding demonstrated a bisatellited chromosome. (**D**) SNP array analysis revealed a 1.5 Mb gain in 22q11.1q11.21 with a copy number of four (**E**) and (**F**) FISH further clarified that the karyotype of fetus 1 was 47,XY,+mar. ish idic(22)(q11.1q11.2)(RP11-958H20++).

In fetus 1, the karyotype of amniocytes revealed to be 47,XY,+mar (Figure 1A). C-banding identified the marker as pseudoisodicentric (psu idic) chromosome, and N-banding demonstrated a bisatellited chromosome (Figure 1B, 1C). Thus, the sSMC was characterized as a pseudoisodicentric and bisatellited chromosome fragment. The pregnant couple had normal karyotypes, indicating that the marker was newly mutated.

To further characterize the origin and gene content of the sSMC, SNP array was performed, the results of which showed a gain of 1.5 Mb in chromosome 22q11.1q11.21 (with a copy number of 4), which contains *SLC25A18*, *IL17RA*, *BID*, *XKR3*, *PEX26*, *CECR1*, *MICAL3*, *CECR2*, *TUBA8*, *USP18*, and *ATP6V1E1 11* Online Mendelian Inheritance in Man (OMIM) genes, of which two (*CECR1* and *CECR2*) are the primary genes associated with cat eye syndrome (CES) (Figure 1D). Subsequently, confirmatory FISH using the BAC probe RP11-958H20 identified four signals on the marker chromosome in all metaphase and interphase cells, which agreed with the CMA findings (Figure 1E, 1F). Based on the SNP array, FISH, and cytogenetic analyses, the final karyotype of case 1 was defined as 47,XY,+idic (22q11.1q11.21) (Table 1).

**Table 1:** Summary of 3 cases presenting de novo sSMC characterized through SNP array and FISH.

**Case**	**Karyotype**	**SNP array Result**	**FISH Result**	**sSMC Morphology**	**Indication of PD**	**Diagnosis**
1	47,XY,+mar dn	Arr[GRCh37]22q11.1q11.21 (16,900,884_18,400,884)×4	47,XN,+mar. ish idic(22)(q11.2)(RP11-958H20++)	psu idic	AMA	CES
2	46,X,+r(X)/45,X	Arr[GRCh37]Xp22.33q11.1 (12,016,549_62,016,549)×1, Xq21.31q28(87,695,881_145,695,881)×1	46,X,+mar. ish r(X)(DXZ1+) [9]/45,X[7]	ring	MSS+	TS
3	45,X/46,X,+idic(Y)	Arr[GRCh37]Yq11.221q11.222 (17,099,010_19,399,010)×2, Yq11.222q11.23(21,035,708_27,135,708)×0	45,X(DXZ1x1,DYZ3x0)[22]/46,X,idic(Y)(q11.2?) 45,X(DXZ1x1,DYZ3x0)[22]/46,X,idic(Y)(q11.2?) (DXZ1x1,DYZ3x2)[2]/ 47,X,idic(Y)(q11.2?)x2(DXZ1x1,DYZ3x4)[1]	idic	MSS+ Abnormal on US	TS

sSMC: small supernumerary marker chromosome; SNP: single nucleotide polymorphism; FISH: fluorescence *in situ* hybridization; AMA: advantaced maternal age; TS: turner syndrome; MSS: maternal serum screening; idic: isodicentric chromosome; US: ultrasound; CES: cat-eye syndrome; PD: prenatal diagnosis

**Figure 2:**
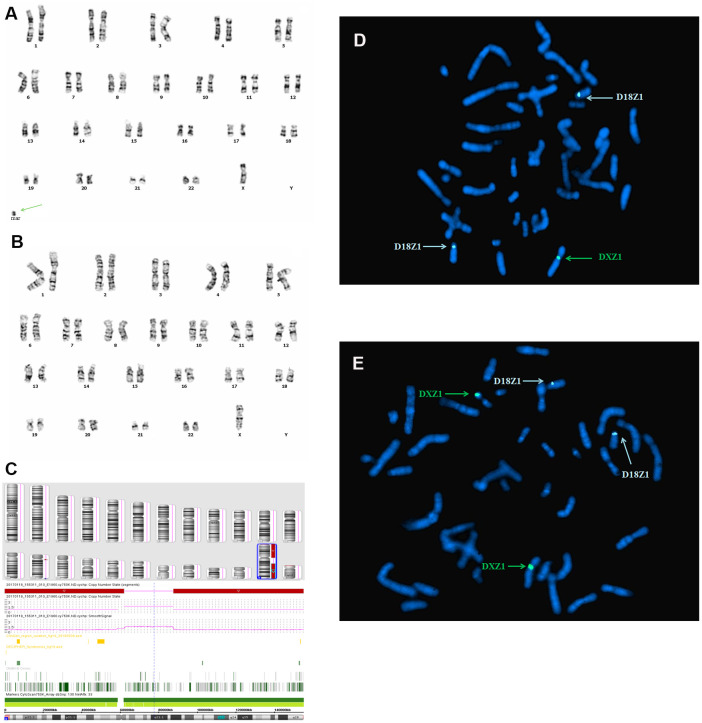
**Karyotype, CMA, and FISH analyses results for fetus 2.** (**A**) and (**B**) a chromosomal karyotyping analysis showed a 45,X mosaic karyotype (46,X,+mar[68]/45,X[32]. The green arrow identifies the sSMC. (**A**) Karyotype analysis revealed 46,X,+mar. (**B**) Karyotype analysis revealed 45,X. (**C**) SNP array analysis revealed a 50 Mb genomic loss at Xp22.33q11.1 (spanning 290 OMIM genes), and a 58 Mb genomic loss at Xq21.31q28 (spanning 296 OMIM genes), including *XIST* gene. (**D**, **E**) Metaphase FISH results using the X, Y, and 18 chromosomal centromeric probes, RB1 and 21S259/D21S341/D21S342 confirmed the karyotype of fetus 2 was 46,X,+mar.ish der(X)r(X)(DXZ1+)[9]/45,X[7].

In case 2, standard chromosomal karyotyping analysis of cultured amniocytes showed a 45,X mosaic karyotype (46,X,+mar[32]/45,X[68] (Figure 2A, 2B). After genetic counseling, the parents of fetus 2 consented to undergo percutaneous umbilical blood sampling. Cytogenetic analysis of umbilical cord blood showed mosaicism 46,X,+mar [22]/45,X[20]. The parental karyotypes were normal. An SNP array analysis revealed a 50 Mb genomic loss at Xp22.33q11.1 (spanning 290 OMIM genes), and a 58 Mb genomic loss at Xq21.31q28 spanning 296 OMIM genes (Figure 2C), including X-inactive specific transcript (*XIST*) gene. Metaphase FISH using the centromeric probes D18Z1, DXZ1, and DYZ3, and a sequence-specific DNA probe for RB1 and 21S259/D21S341/D21S342, which are located on 21q22.13, confirmed the SNP array findings (Figure 2D, 2E). Furthermore, FISH analysis identified the karyotype as 46,X,+mar.ish r(X)(DXZ1+)[8]/45,X[6]. By combining the CMA and FISH results, the marker chromosome was determined to be derived from the ring X chromosome, encompassing *XIST* gene, thus, the karyotype of fetus 2 was defined as 45,X/46,X,r(X) (p11.1q21.31) (Table 1).

**Figure 3:**
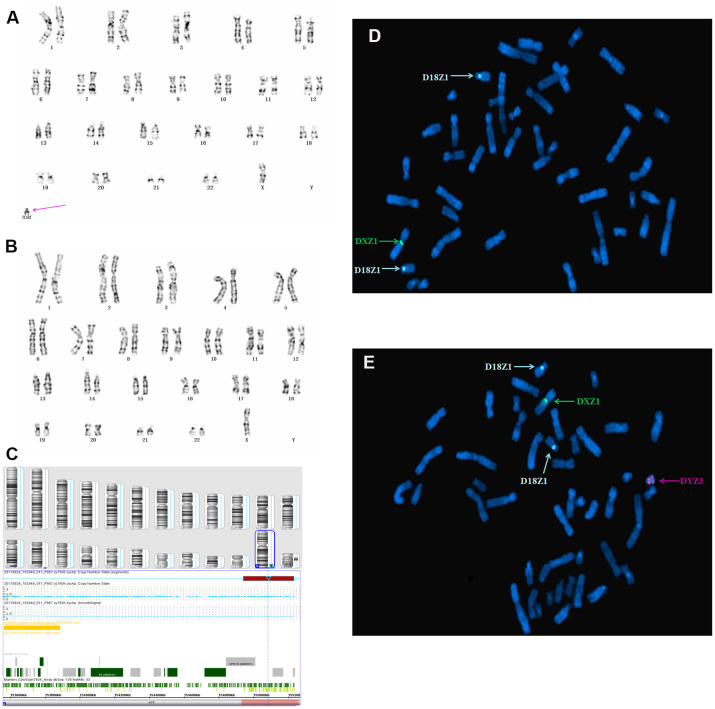
**Karyotype, CMA, and FISH analyses results for fetus 3.** The purple arrow identifies the sSMC. (**A**) and (**B**) conventional karyotype analysis revealed 45,X[62]/46,X,+mar[9]. (**C**) SNP array analysis revealed a 2.3 Mb genomic gain in Yq11.221q11.222 and a 6.1 Mb genomic loss in q11.222q11.23, spanning 15 OMIM genes, including *HSFY*1, *PRY*, *DAZ*1, *AZFb,* and *AZFc*. (**D**, **E**) Metaphase FISH analysis using X, Y, and 18 chromosomal centromeric probes revealed the karyotype of fetus 3 to be 45,X(DXZ1×1, DYZ3×0)[22]/46,X,idic(Y)(q11.2?) (DYZ3×2,DXZ1×1)[2]/47,X,idic(Y)(q11.2?)×2(DYZ3×4,DXZ1×1)[1].

In case 3, cytogenetic analysis using cultured amniocytes revealed that the karyotype of fetus 3 was 45,X[62]/46,X,+mar[9]. Moreover, 12.7% cells were found to harbor an sSMC and 87.3% of cells had 45,X (Figure 3A, 3B). Subsequent SNP array analysis of amniocytes revealed a 2.3 Mb genomic gain in Yq11.221q11.222 (encompassing one OMIM gene) and a 6.1 Mb genomic loss in Yq11.222q11.23, spanning 18 OMIM genes and encompassing *HSFY1*, *PRY,* *DAZ*1, *AZFb*, and *AZFc*, which are associated with azoospermia, oligospermia, and infertility (Figure 3C). Further FISH analysis using X, Y, and 18 chromosomal centromeric probes defined the karyotype of fetus 3 as 45,X(DXZ1×1, DYZ3×0)[22]/46,X,idic(Y)(q11.2?) (DXZ1×1, DYZ3×2)[2]/47,X,idic(Y)(q11.2?)×2(DXZ 1×1, DYZ3×4)[1]), which generally agreed with the SNP array results (Table 1).

### Pregnancy outcomes and follow-up study

Three *de novo* sSMCs were identified in three pregnancies. One pregnancy was continued, whereas two pregnancies, one with Turner syndrome (TS) and one with CES, were terminated by delivery through induced labor (Table 1, 2).

**Table 2:** Genetic detection results, ultrasound findings and clinical information for the 3 fetuses.

**Case**	**Maternal Age, Y**	**History**	**Gestation at Diagnosis, wk**	**Fetal Specimen**	**Karyotype**	**Size, Mb**	**Ultrasound Findings**	**Pregnancy Outcome**	**Other Findings at Birth**
1	38	G4P1	19^+1^	AF	47,XY,+mar dn	1.5	Dominant right heart, Ventricular septal defect, Dysplasia of aorta, Polyhydramnios, LIEF, SUA	CTP Cesarean section Died at 10 days of life	Left ear canal atresia, Bilateral preauricular skin tags, Flattened nasal bridge Low-set ear Hypertelorism
2	27	G2P0	18^+4^	AF	46,X,+mar[32]/45,X[68]	50	Patent foramen ovale,	TOP 24^+2^ wk	NA
			22	CB	46,X,+mar[22]/45,X[20]	58	Aortic stenosis,		
3	33	G2P1	18+1	AF	45,X[62]/46,X,+mar[9]	2.3/6.1	Fetal tricuspid regurgitation, Broad inner diameter of the right pulmonary artery	TOP 23 wk	Normal

Dn: de novo; TOP: termination of pregnancy; sSMC: small supernumerary marker chromosome; CTP: continue the pregnancy; TOP: terminate the pregnancy; AF: amniotic fluid; CB: cord blood; LIEF: left intracardiac echogenic focus; SUA: single umbilical artery; NA: not available

### Case 1

After adequate genetic counseling, despite the ultrasound abnormalities and the unfavorable prognosis for fetus 1 of sSMCs associated with CES, the parents accepted the risk of an unpredictable degree of CES in the child and the pregnancy continued to term. At 38 weeks of gestation, a phenotypically abnormal boy was born, weighing 2900 g at birth, and Apgar scores of 8-9-10. The woman denied taking any teratogenic medicines or having any illness during the pregnancy. In addition to the prenatal findings, postnatal examination revealed bilateral preauricular skin tags, hypertelorism, left ear canal atresia, anal atresia, flattened nasal bridge, and bilateral low-set ear. Ophthalmological examination showed no anomaly; however, due to severe lung hypoplasia, the boy died at home on day 10 of life. Permission for an autopsy was not granted (Table 2).

### Case 2

Normal female genitalia were observed sonographically. Given the chromosomal abnormality and the adverse prognosis, labor was induced at another hospital at 24^+2^ weeks of gestation after adequate genetic counseling. Thus, clinical data were not available (Table 2).

### Case 3

Normal male genitalia were observed sonographically. In the context of cardiovascular and chromosomal abnormalities, the pregnancy was electively terminated after the karyotype, SNP array, and FISH results were disclosed. A phenotypically normal male, weighing 250 g and showing no external malformations, was delivered through induced labor. Further post-mortem studies were declined. Thus, autopsy data were not available (Table 2).

## DISCUSSION

Generally, conventional cytogenetic banding can only detect sSMCs, while determining the precise origin of the sSMC is challenging. sSMC frequencies have been reported to differ according to the population group studied [2]. Moreover, patients with sSMCs vary greatly as the phenotypic differences are closely related to chromosome origin, gene content, size, degree of mosaicism, presence of uniparental disomy, and distribution pattern of the sSMC in different tissues. Considering that the risk of phenotypic abnormalities in fetuses with sSMCs is 13% [5], the precise characterization of sSMCs is crucial for prenatal diagnosis and genetic counseling. However, identifying the genotype–phenotype correlations is challenging in prenatal diagnosis, particularly for *de novo* sSMCs. Nevertheless, FISH has proven essential for this purpose, even with the recent boom in microarray techniques. Furthermore, SNP array is routinely applied for the detection of chromosomal submicroscopic deletion/duplication, UPD as well as for prenatal identification of sSMCs [6]. In fact, it serves to supplement cytogenetic analysis in genetic counseling, as well as in evaluating the prognosis of fetuses with sSMCs. Hence, SNP arrays combined with FISH technologies can greatly reduce prognostic uncertainty while providing critical information that helps couples to choose whether to continue or terminate the pregnancies.

In the current study, abnormal phenotypes were observed for the three fetuses and a mosaic condition was identified in two fetuses with a TS karyotype. Of the three cases with pathogenic CNVs detected by SNP arrays, in terms of morphology, the sSMCs were isodicentric bisatellited, ring, and isodicentric marker chromosomes.

CES commonly results from a partial tetrasomy of 22p, and derives from a supernumerary dicentric and bisatellite sSMC, as well as chromosome 22q11 inverse duplication [7]. Its classical manifestations include iris colobomas, anal malformations, and ear anomalies triad. However, although nearly 40% of CES patients develop this classical triad of symptoms, [8], this syndrome is associated with highly variable phenotypes, ranging from nearly normal to severe multiple malformations [9]. Rare clinical presentation such as anatomic insufficiency of the chest and spleen [10], hemifacial macrosomia [11], Müllerian agenesis [12], and congenital diaphragmatic hernia [13] have been reported. According to the location of the breakpoint, CES is divided into two types: type I CES does not involve the critical region of CES and the breakpoints of deletion and/or duplication are located in the proximal region of 22q11; Type II CES involves one breakpoint (asymmetric type, IIa) or two breakpoints (symmetric type, IIb) in the distal region, encompassing one or two copies of DiGeorge syndrome and CES critical region [14]. Furthermore, partial trisomy of chromosome 22, as well as 22q11.21 intrachromosomal triplication, can reportedly cause the CES phenotypes [15].

Crolla et al. [16] reported that 68% of all sSMCs originate from proximal centromeric chromosomes, of which sSMC(22) accounts for 13%. The mechanism responsible for sSMC(22) involves an exchange error of one pair of homologous chromosome monomers during meiosis, forming a U-type exchange, and constituting partial tetraploid of 22p karyotype. In this study, the sSMC of fetus 1 was identified as a pseudodicentromere and bisatellite chromosome fragment by C-banding and N-banding (Figure 1A–1C), respectively, indicating its origin to be of a proximal centromeric chromosome. By applying SNP array gene chip technology combined with FISH verification, the specific sSMC was determined to originate from chromosome 22, and the tetraploid portions of 22q11.1q11.21, leading to CES. The karyotype of fetus 1 was further defined as 47,XY,+mar.ish idic(22)(q11.1q11.2)(RP11-958H20++) by subsequent FISH analysis (Figure 1E–1F). The SNP array analysis shows a 1.7 Mb gain at 22q11.1q11.21, with the copy number of 4, thus causing type I CES.

Considering that prenatal ultrasound screening and serological tests are unable to predict CES, as the main features of CES are nonspecific, prenatal CES is often diagnosed by chance. For instance, in case 1, the indication for prenatal diagnosis was advanced maternal age. Approximately 50% of CES patients develop iris coloboma defect [8–10]. Furthermore, besides facial dysmorphism, impaired ocular motility and cryptorchidism occur in 25–76% and 24% of CES patients, respectively. In fetus 1, prenatal ultrasound revealed fetal dominant right heart, ventricular septal defect, left ventricular echogenic focus, aortic dysplasia, single umbilical artery, polyhydramnios, hypertelorism, bilateral preauricular skin tags, left ear canal atresia, and imperforate anus. Postpartum physical examination revealed that fetus 1 presented anal and ear anomalies, which further confirmed the results of prenatal diagnosis.

SNP array results further identified arr[hg19] 22q11.1q11.21 (16,888,899 _18,649,190)x4 dn. SNP array analysis showed a 1.7 Mb duplication at 22q11.1q11.21 spanning 11 OMIM genes, including USP18, SLC25A18, XKR3, CECR2, BID, IL17RA, PEX26, CECR1, ATP6V1E1, TUBA8, and MICAL3, among these genes, CECR1 and CECR2 are key genes that contribute to phenotypic changes associated with the duplication in CES [17]. CECR1 is highly expressed in the heart outflow, the atrium, the face, and VII/VIII cranial nerve ganglia, and is associated with facial malformation and cardiac defects [18]. It is also associated with heart and kidney defects [19]. CECR2, containing leucine ziplines and bromine domains, is considered to be a chromatin remodeling gene. Most CES patients present with eye and ear abnormalities due to the overexpression of CECR2, which affects the development of the brain, eyes, and ears [20]. Meanwhile, MICAI3 and TUBA8, are responsible for cytoskeletal structure during neuronal migration, and BID regulates cell cycle arrest and apoptosis. Hence, the overexpression of these three genes may be associated with nerve injury in CES patients [21].

It is reported that gain of the CES critical region (CESCR) (encompassing from centromere to the D22S57 [14]) is associated with congenital heart disease, as well as anorectal, urogenital, and preauricular anomalies. Fetus 1 manifested anorectal, heart, and preauricular anomalies, the study [15] agreed with those reported by Meas [22] as well as those of our case 1. Hence, duplication of these different genes in fetus 1 may have accounted for the observed dominant right heart, ventricular septal defect, dysplasia of the aorta, polyhydramnios, strong echo of the left ventricle, and hypertelorism. However, prenatal ultrasound did not detect eye or kidney anomalies. Multiple cases have been reported involving candidate CECR2 gene without ocular defects [23, 24]. Therefore, except for the gene dose effect, the interaction of upstream and downstream regulating elements of the above-mentioned genes, as well as gene-environment interactions, may be responsible for the penetrance and phenotype differences associated with CES.

### sSMC in Turner syndrome case

TS occurs in 1.76 per 1,000 female fetuses upon mid-trimester amniocentesis [25], and the incidence of sSMCs in TS (sSMC^T^) is 3.08% [26]. sSMC^T^ carriers primarily have a 45,X/46,X,+mar karyotype. Meanwhile, sSMC^T^ has been associated with three morphologies: diccentromeric (dic), ring chromosome (r), and small chromosomal segments with centromeres (min). Nearly all sSMC^T^s are confirmed to be a derivative of chromosome X or Y, and approximately 72.6% originate from Y, while most sSMC^T^(Y) are from isodicentric chromosomes. A further 27% originate from the X chromosome with a majority of sSMC^T^s(X) determined to be ring chromosomes [27]. The remaining 0.4% are derived from autosomal chromosomes [28] and may be responsible for the TS phenotypes or gonadal dysgenesis.

The correlation analysis between sSMCs and clinical phenotypes not only contributes to the elucidation of the mechanism and genetic effect of sSMCs, but also has important clinical implications for the genetic consultation and treatment selection of TS fetuses. Phenotypes of individuals carrying mos45,X/46,X,+mar differ depending on the gene content, mosaicism proportion, size, X chromosome breakpoint location, and *XIST*-mediated silencing. Patients carrying sSMC^T^(X) may present a mild variant TS phenotypes if sSMC^T^(X) is inactivated [29]. Meanwhile, patients with a 45,X/46,XY karyotype are at significantly increased risk for gonadoblastoma. Both gonadoblastoma and *in situ* carcinoma can progress to non-invasive or invasive malignant germ cell tumors [30]. Some researchers consider that the *GBY* on Yp11-q11 may lead to the occurrence of malignant gonadal tumors [31, 32]. In our present study, cases 2 and 3 with sSMC^T^ were detected prenatally (Table 1). Since the prognosis of patients carrying different chromosome sources of sSMC^T^ vary greatly, once TS with sSMC^T^ is diagnosed prenatally it is necessary to further characterize the origin and morphology of sSMC^T^ using molecular genetic technologies.

In case 2, karyotyping analysis of amniocytes revealed that the fetus was 32% mosaic (46,X,+mar[32]/ 45,X[68] (Figure 2). SNP array revealed that the derivate corresponded to the Xp11.1-q21.31 region, which contains the *XIST* gene (Xq13.2), and its preferential inactivation is possible. Subsequently, FISH (VYSIS, Inc) analysis using a DXZ1 probe indicated that the marker chromosome originates from ring X chromosome [r(X)]. As a result, the karyotype was defined as mos 45,X[68]/46,X,r(X)(p11.1q21.31)[32], which is diagnosed as TS. The r(X) with *XIST* expression can cause random X inactivation. However, the abnormal severe TS phenotypes may be caused by the inactivation bias of r(X). The X inactivation test was not carried out for fetus 2 as per the mother’s instructions. Fetus 2 had no major structural anomalies apart from hydrops fetalis based on the SNP array result. We, therefore, postulate that *XIST* may mediate sSMC^T^(X) inactivation in this specific case.

Supernumerary ring chromosomes constitute approximately 10% of SMCs [33]. Abnormally sized r(X) accounts for 5% of TS patients [34]. In fact, most individuals with larger r(X) tend to have TS phenotypes, without mental retardation. However, a severe TS phenotypes (mental retardation/developmental delay) may also be due to the inactivation bias of X chromosome [35]. Most of these patients require therapeutic intervention for infertility. However, Yuge et al.[36, 37] reported a normal pregnancy, that did not require therapeutic intervention for infertility, in a patient diagnosed with TS that had mos 45,X/46,X,r(X) karyotype.

It is impossible to determine the mosaicism proportion in different endoderm/ /mesoderm/ectoderm tissues, however, the relatively high proportion of amniocytes with an abnormal karyotype in case 2 (32%) could be a poor prognostic sign. Moreover, the haploinsufficiency effect of SHOX gene at Xp22.3 in fetus 2 may lead to early fusion of distal limb growth as well as short stature. The female ovarian function maintenance region is primarily located at Xp11 and Xq13-q26, while deletion in Xq28 can cause premature ovarian failure and infertility, of which, the involved genes POF1 and POF2 are located in Xq26-q28 and Xq13.3, respectively [37]. Therefore, we propose that fetus 2 might present with short stature, primary amenorrhea, premature ovarian failure, uterine dysplasia, breast dysplasia, and infertility since puberty. Finally, the pregnancy was terminated electively after genetic counseling.

In case 3, the pregnant woman performed amniocentesis due to high risk of serological Down syndrome screening in the second trimester, meanwhile, prenatal ultrasound showed fetal tricuspid regurgitation with broad inner diameter of the right pulmonary artery. Karyotype analysis of amniocytes showed 45,X[62]/46,X,+mar[9], of which, 45,X cell lines accounted for 87.3%. The family history was unremarkable. Subsequent SNP array analysis of amniocytes revealed a 2.3 Mb genomic gain in Yq11.221q11.222 (encompassing one OMIM gene) and a 6.1 Mb genomic loss in q11.222q11.23 (containing AZFb and AZFc) encompassing 20 OMIM genes, including HSFY1, PRY, and DAZ1, which can cause oligospermia and infertility in men (Figure 3C). In addition, to clarify the origin and morphology of the sSMC, FISH assay was carried out using DYZ3 (red) centromeric probe located at Yp11.1-q11.1. In the metaphase cells, four red signals were detected on the sSMC, indicating that the sSMC was isodicentromeric of Yp chromosome. This provides a strong basis for elucidating the recombination mechanism of sSMC. Ultimately, the sSMC was determined as idic (Y)(q11.2).

Although the mechanism of idic(Y) formation remains unknown, Beaulieu et al. [38–40] studied patients with idic(Y) karyotype, and proposed that Y chromosome may not be separated during phase I and II of meiosis as well as sister chromosome monomer during mitosis, which is an important mechanism for idic(Y) formation. Moreover, Mekkawy et al. [41] suggested that breakpoints of idic(Y) often occur in regions of the repeat sequence, and considered that the special structure in these regions could easily cause non-allelic exchange recombination within chromosomes, resulting in the deletion, inversion, repetition, and idic(Y), and subsequent deletion of sperm-related genes.

We, therefore, speculated that fetus 3 carrying SMC(Y), encompassing *GBY*, had an increased risk of gonadal tumor occurrence. Hence, should the couple have opted to continue the pregnancy, the fetus would have to be followed-up closely. Further karyotype analysis performed on skin and gonad tissue would also be required and dysplastic gonad would have to be removed laparoscopically as soon as possible to avoid tumorigenesis in the case of the presence of Y chromosome.

Isodicentric Y chromosomes are unstable and are generally lost during mitosis, producing a 45, X cell line accounting for majority proportion, thus forming a mosaic 45, X/46, X, Idic(Y) [42]. The karyotype 45,X/46,X,idic(Y) is rarely detected prenatally and the phenotypes of these patients, ranging from TS to sexual developmental disorder, sex ambiguity to azoospermia, and mental retardation, may be influenced by the location of Y chromosome breakpoints, the proportion of abnormal cell lines, and whether the SRY gene is lost [43–45]. In addition, clinical phenotypes are not necessarily related to the proportion of the two cell lines, since the karyotype in the fetal sex gland system may differ entirely from that in amniotic fluid cells. Thus, the clinical manifestations of patients carrying mos45,X/idic(Y) are congenital gonadal dysplasia and genital malformation [46]. Moreover, AZFb, AZFd, and AZFc regions are absent in patients with idic(Y), resulting in spermatogenesis disorder, and potentially azoospermia. Willis et al. [47] demonstrated that most individuals diagnosed as idic(Y) prenatally, will be male with a normal phenotype. However, affected individuals may be at risk for growth deficiency, developmental delay, and infertility [43–44]. We detected a breakpoint on Y(q11.2) and a large deletion spanning many genes in case 3. Some of these genes, including PRY, HSFY1, and DAZ, are strongly linked to male infertility [48, 49]. Specifically, the HSF family members, HSF1 and HSF2, are likely to play important roles in human spermatogenesis as some azoospermia patients harbor deletion of HSFY on AZFb [49, 50]. In fetuses with 45,X/46,X idic(Y) karyotype, the 45,X cell line may play a more significant role in sexual differentiation, independent of the percentage of abnormal Y cell lines in the prenatal sample [47]. However, to accurately predict its future phenotypes, long-term longitudinal follow-up studies on the fetus carrying identical karyotypes are necessary. Given that fetus 3 had a high risk for gonadoblastoma and infertility, after adequate genetic counseling and informed consent, the pregnancy was terminated.

In conclusion, phenotype–karyotype correlations of sSMCs pose a major challenge in prenatal diagnosis. Combined use of cytogenetics, FISH, and CMA can clarify the origin and pathogenicity of sSMCs, which is not only helpful to characterize sSMCs formation mechanisms and genetic effects, but also has important clinical significance for fetal genetic consultation, treatment selection, and ultimately, parental decision making.

## MATERIALS AND METHODS

### Case presentation

### Case 1

A 37-year-old, gravida 4, para 1, pregnant woman underwent amniocentesis at 19^+1^ weeks of gestation due to advanced maternal age. She and her husband were healthy and nonconsanguineous, and they had a 9-year-old healthy son. There was no family history of congenital malformations. Prenatal ultrasound examination at 20 weeks of gestation revealed fetal right dominant heart, ventricular septal defect, aortic dysplasia, left ventricular punctate echogenicity, and single umbilical artery (Table 2). No other abnormalities were observed.

### Case 2

A 27-year-old, gravida 2, para 0, pregnant woman was referred for amniocentesis at 18^+4^ weeks of gestation for a positive first-trimester maternal serum screening for Down syndrome (pregnancy-associated plasma protein A: 524 mU/L[0.365 MOM (0.45–2.0)], and free β-human chorionic gonadotrophin (hCG): 147 ng/mL[1.42 MOM (0.25–2.0)), screening was suggestive of increased risk for trisomy 21 (1/198). Cytogenetic analysis showed a 45,X mosaicism karyotype (46,X,+mar[32]/45,X[68]). After adequate genetic counseling, the parents of fetus 2 consented to undergo percutaneous umbilical blood sampling. Cytogenetic analysis using umbilical cord blood showed mosaicism 46,X,+mar[22]/45,X[20]. The pregnancy was uncomplicated. There was no family history of congenital anomalies. A second ultrasound examination at 22 weeks of gestation revealed patent foramen ovale and aortic stenosis (Table 2). Normal female genitalia were observed sonographically.

### Case 3

A 30-year-old, gravida 2, para 1, pregnant woman was referred for amniocentesis due to abnormal triple marker screening in the second trimester, screening suggestive of a high risk of Down syndrome 1:204. Maternal serum uE3 was 3.97 nmol/L(0.56 MOM), α-fetoprotein was 43.3 U/mL (0.93 MOM), and free β-hCG was 32.2 ng/mL (2.62 MOM). Amniocentesis was performed at 18^+1^ weeks of gestation. There was no other relevant family history and the couple was nonconsanguineous. A detailed fetal ultrasound at 22^+5^ weeks of gestation revealed fetal tricuspid regurgitation, as well as a broad inner diameter of the right pulmonary artery. No other malformations were detected (Table 2).

### Chromosomal karyotyping

Amniotic fluid or fetal cord blood sample was obtained according to the invasive procedure protocol. Routine cytogenetic analysis by G-banding (C/NOR-banding when necessary) technique was performed according to standard laboratory protocols and ISCN 2016. Briefly, 20 mL of amniotic fluid was collected and subjected to *in situ* amniocyte culture. Parental blood samples were also collected for cytogenetic analyses. Amniocyte *in situ* culture and harvest, as well as G-banding were performed. Fifteen primary colonies were examined. If the available number of the primary colonies was fewer than 15, a total of 20 cells from both primary and trypsinized cultures were examined.

### Single nucleotide polymorphism arrays

SNP arrays constitute one type of CMA technology capable of detecting genome wide CNVs. Here, a high-resolution SNP array was initially performed on three fetuses carrying sSMCs using an Affymetrix array (CytoScan® 750 K; Affymetrix/Thermo Fisher Scientific, Santa Clara, CA, USA) according to manufacturer’s instructions. The procedure included genomic DNA extraction, digestion and ligation, PCR amplification, PCR product purification, quantification and fragmentation, labeling, array hybridization, washing, and scanning, and the results were analyzed with CHAS software (Affymetrix/Thermo Fisher Scientific) using annotations of the genome version GRCh37. The reporting threshold was set at gains or losses ≥ 400 kb and loss of heterozygosity ≥ 10 Mb. For the interpretation of these results, our local database and the following public database were used: DGV (http://projects.tcag.ca/variation/), Cytogenomics Array Group CNV Database (http://www.cagdb.org/), Database of Chromosomal Imbalance and Phenotype in Humans using Ensembl Resources database (DECIPHER, http://decipher.sanger.ac.uk/), Online Mendelian Inheritance in Man (OMIM, http://www.omim.org). Parental analysis was performed to interpret VOUS when necessary. sSMCs were initially characterized using a SNP array and then were further confirmed by FISH.

### Fluorescence *in situ* hybridization

FISH was carried out on the three cases. The probes were selected based on the CNVs detected by the SNP array. Commercial probes were used to target chromosomes 13/21, 14/22, 15/16, and 18, the chromosome X α-satellite centromere (DXZ1 at Xp11.1-q11.1), the Y α-satellite region (DYZ3 at Yp11.1-q11.1), and BAC clone probe RP11-958H20 at 22q11.1-22q11.2. FISH was performed on interphase/metaphase amniocytes according to the manufacturer’s instructions (Vysis, Downers Grove, IL, USA).

### Data availability statement

The data that support the findings of this study are available from the corresponding author.

### Statement of ethics

The study was approved by the ethics boards of Fujian Provincial Maternity and Children’s Hospital (No.12, No.11, and No.10), Each patient received written informed consent for participation. All procedures were performed in accordance with the Declaration of Helsinki.
